# Domains associated with successful quality improvement in healthcare – a nationwide case study

**DOI:** 10.1186/s12913-017-2454-2

**Published:** 2017-09-13

**Authors:** Aleidis Skard Brandrud, Bjørnar Nyen, Per Hjortdahl, Leiv Sandvik, Gro Sævil Helljesen Haldorsen, Maria Bergli, Eugene C. Nelson, Michael Bretthauer

**Affiliations:** 1 Quality Department, Vestre Viken Health Trust, Wergelandsgate 10, Postbox 800, 3004 Drammen, Norway; 2 Municipality of Porsgrunn, Porstbox 128, N-3901 Porsgrunn, Norway; 3 Department of Family Medicine, Faculty of Medicine, University of Oslo, PO Box 1130, Blindern, NO-0318 Oslo, Norway; 4 Oslo Center for Biostatistics and Epidemiology, Research support Services, Oslo University Hospital, Sogn Arena, Klaus Torgaards vei 3, 0372 Oslo, Norway; 5 South Eastern Norway Regional Health Authority, PO Box 404, 2303 Hamar, Norway; 6 The Dartmouth Institute for Health Policy and Clinical Practice, Geisel School of Medicine at Dartmouth, 30 Lafayette Street, Lebanon, NH USA; 7 Department of Health and Society, Faculty of Medicine, University of Oslo, PO Box 1130, Blindern, NO-0318 Oslo, Norway

**Keywords:** Quality improvement, Learning collaboratives, Continual improvement, Conditions for change, Context

## Abstract

**Background:**

There is a distinct difference between what we know and what we do in healthcare: a gap that is impairing the quality of the care and increasing the costs. Quality improvement efforts have been made worldwide by learning collaboratives, based on recognized continual improvement theory with limited scientific evidence. The present study of 132 quality improvement projects in Norway explores the conditions for improvement from the perspectives of the frontline healthcare professionals, and evaluates the effectiveness of the continual improvement method.

**Methods:**

An instrument with 25 questions was developed on prior focus group interviews with improvement project members who identified features that may promote or inhibit improvement. The questionnaire was sent to 189 improvement projects initiated by the Norwegian Medical Association, and responded by 70% (132) of the improvement teams. A sub study of their final reports by a validated instrument, made us able to identify the successful projects and compare their assessments with the assessments of the other projects. A factor analysis with Varimax rotation of the 25 questions identified five domains. A multivariate regression analysis was used to evaluate the association with successful quality improvements.

**Results:**

Two of the five domains were associated with success: *Measurement and Guidance* (*p* = 0.011), and *Professional environment* (*p* = 0.015). The organizational leadership domain was not associated with successful quality improvements (*p* = 0.26).

**Conclusion:**

Our findings suggest that quality improvement projects with good guidance and focus on measurement for improvement have increased likelihood of success. The variables in these two domains are aligned with improvement theory and confirm the effectiveness of the continual improvement method provided by the learning collaborative. High performing professional environments successfully engaged in patient-centered quality improvement if they had access to: (a) *knowledge of best practice* provided by professional subject matter experts, (b) *knowledge of current practice* provided by simple measurement methods, assisted by (c) *improvement knowledge* experts who provided useful guidance on measurement, and made the team able to organize the improvement efforts well in spite of the difficult resource situation (time and personnel). Our findings may be used by healthcare organizations to develop effective infrastructure to support improvement and to create the conditions for making quality and safety improvement a part of everyone’s job.

**Electronic supplementary material:**

The online version of this article (doi:10.1186/s12913-017-2454-2) contains supplementary material, which is available to authorized users.

## Background

Healthcare is suffering from serious unsolved problems that are threatening lives, increasing costs, and making the care unpredictable to the patient [1–7]. Improvement of quality in health care, is probably one of the greatest challenges of modern healthcare leadership. Quality improvement strategies sometimes fail to focus the changes on clinical, patient oriented improvements, and to involve the frontline healthcare professionals at an early stage of the change process [8–10].

The role of qualified *improvement guidance* has received little attention in the quality improvement literature [11–15]. A recent analysis of 35 systematic reviews explored the influence of context on the effectiveness of different quality improvement strategies. Improvement guidance was not found among a broad range of associated contextual factors that contribute to successful improvement. The analysis organized the findings based on the Model for Understanding Success in Quality (MUSIQ) model [16, 17]. The MUSIQ model itself was based on a systematic review that included continual improvement interventions, but did *not cover* the role of improvement knowledge guidance [14, 17]. A cluster-randomized trial aimed to compare clinic-level coaching with other learning collaborative components, found coaching to be equally effective with interest circle calls (group telephone conferences) in achieving clinical outcome improvements, but coaching was more cost-effective [18]. Godfrey did also find positive effects of systematic clinic-level coaching [19, 20].

In a case study of 182 improvement teams Strating found that creating *measurable targets* is a crucial task in quality improvement [21]. In a systematic review of quality measurement*.* Thor et al. found statistical process control (SPC), to be a useful method for those who mastered the technique [22]. This underscores the importance of good measurement guidance.

Many healthcare organizations do not have a *basic infrastructure* to support improvement, and contextual factors generally receive scant attention in the current literature on quality improvement strategies [13, 14, 16, 22, 23]. Kringos et al. found that the availability and functionality of information technology and facilitated data collection improved the effectiveness of quality improvement intervention, as well as the involvement of multidisciplinary improvement teams [16].

Little evidence is found that *leadership support* is associated with successful quality improvement [24–26]. This may be typical for external initiated learning collaboratives, because we found a few studies where the frontline leaders have been directly included in the project planning and improvement guidance, with a positive leadership influence on the effectiveness of the improvement efforts [16, 18–20].

Since 1994, and in spite of a *limited underpinning of scientific evidence*, the continual improvement method has been spread worldwide by thousands of improvement collaboratives [13, 27–29]. Relatively little of that work is reported in the biomedical literature [30]. Systematic reviews and single studies of quality improvement efforts that are reported, indicate that a systematic and knowledge based approach is not enough to succeed without the presence of certain conditions for improvement, also described as context factors [13, 14, 16, 31]. To meet these challenges, additional improvement approaches, including instruments for evaluating the underlying conditions for improvement, have been described [12, 17, 32]. In 2004 a systematic review recommended further research on factors that tend to produce adoptable changes in healthcare organizations [33]. A recent umbrella review of 35 systematic reviews of the influence of context factors on the effectiveness of (any) quality improvement intervention recommend further research to report the context factors in a systematic way to better appreciate their relative importance [16].

*The present study explores the conditions for improvement* in the context of 189 Norwegian clinical improvement projects initiated by the learning collaboratives of The Norwegian Medical Association. We asked participating clinicians to identify factors that may promote or inhibit quality improvement. Referring to the studies above, two of the unanswered questions are (1) “*What combination of what factors tend to produce “adoptable” improvement innovations*?” [33]. (2) *How is the effectiveness of the continual improvement method?* (The continual improvement method is described in Additional file 1: Supplement 1). The purpose is to identify domains associated with success, as this knowledge may be used to develop an infrastructure and culture that promotes continual improvement in healthcare, without the help from a learning collaborative.

## Methods

### Summary

The method of the present study had four steps. *First* we developed a questionnaire for improvement teams. The instrument was based on a qualitative study of the conditions for change among 19 participants of the learning collaboratives of the Norwegian Medical Association (Sub study I, published in 2011) [23]. *Second,* we submitted the questionnaire to the 189 improvement teams of the same learning collaboratives. *Third*, we analyzed the data by comparing the reported conditions for improvement in the organizations of the successful projects versus the other (comparator) projects. We already knew the success level of the 189 projects from the validation of a Change Process and Outcome Scale instrument which was published in 2015 (Sub study II) [32].

### The learning collaboratives

Between 1998 and 2011 The Norwegian Medical Association sponsored eight hospital related *improvement collaboratives* to support quality and safety improvement efforts in clinical environments (Table 1). The improvement collaboratives were based on the Breakthrough Series model of the Institute for Healthcare Improvement, aiming to accelerate improvement beyond what had been achieved by traditional educational approaches [34]. The model has two dimensions: the *learning collaborative method* (national level), and the *systematic approach to continual improvement* (organizational level) [11, 22, 35–42] (described in Additional file 1: Supplement 1). *Each collaborative* lasted from 6 to 9 months and engaged clinicians from 15 to 30 healthcare organizations who met to learn from each other and from recognized experts in specified topic areas (Table 1). The participating improvement teams sent 2–4 representatives from different disciplines involved in the topic (at least one physician) to three collaborative learning sessions, where the relevant subject matter experts of the collaborative (medicine, nursing, psychology, etc.) demonstrated the quality gaps within the topic to the participants. A team of 10–15 improvement knowledge experts (coaches) guided the improvement teams at, and in between the learning sessions.

**Table 1:** The eight hospital-related improvement collaboratives of the Norwegian Medical Association

Year	Topic	Number of projects	Examples of the measurable aims of the single projects within each Improvement collaborative
1999	Cesarean Section	23	Reduce the cesarean sectio rate to para 0 by 20%.
2000	Intensive Care	15	Reduce the duration of mechanical ventilation by 20% by an optimization of the sedation; reduce length of ICU stay by 10%.
2001	The Use of Restraints	18	Reduce the use of mechanical restrains in psychiatric therapy by 25%.
2003	Serious Affective Disorders	23	Reduce the MADRS score by 50% and the length of stay by 50%.
2004	ADHD	33	Reduce the time from admission to diagnosis by 30%
2005	Quality & Efficacy in Psychiatric Outpatient Clinics	30	Increase the quality of the preliminary feedback to the primary care physician by 50%
2006	Substance Abuse	24	Increase the quality of the admission process by 50%
2011	Early Intervention in Psychiatric Disorders	23	Reduce the days between first time referrals by 50%, and the age of the patients by 25%.

### Instrument development

The questionnaire was developed to identify the activities and conditions associated with successful quality improvement initiatives, and to study the effectiveness of the continual improvement method (Additional file 1: Supplement 1). The instrument and its development is described in details in Additional file 1: Supplement 2.

### Sub study I

The first sub study was published in 2011 [23], and provided us with a large amount of relevant comments, and enabled us to develop a validated questionnaire reflecting the most interesting conditions for change reported by clinicians telling their improvement project stories from their own organizations, after participating in a learning collaborative of the Norwegian medical association.

### Data collection

The questionnaire was submitted to former improvement team leaders between 2 and 4 years after the end of each improvement collaborative. We had access to their e-mail addresses from the improvement collaborative participant list. A link to an on-line questionnaire was e-mailed to the improvement team leaders. They were asked to think back on their improvement project and the promoting and inhibiting conditions for quality improvement that they encountered, and to show their level of agreement with the focus group comments that were included in the questionnaire.

If a Word-version of the questionnaire was preferred, the respondents returned their filled-in questionnaires by e-mail or “surface mail”. In cases of non-response from team leaders, we contacted other participants from the same team. In 36% of the teams, late responses lead to more than one response from the same team. Because the responses from team members mostly reflected different professions, and the inter-rater reliability of the same team ranged from poor to strong, we decided to let each team be represented by the average ratings of its responding members.

### Project evaluation

This study is neither an experiment, nor a study of the experiments of others, aiming to bring evidence to the success of the projects in our material. This is a study of the conditions for making successful changes. The aim is to learn from healthcare professionals in the improvement teams of those projects who have been able to document improvements.

### Sub study II

The second sub study was published in 2015 [32]. Seven improvement experts from different healthcare professions alternated in participating in a four-person review team. The reviewers were two physicians (BN and TSH), three nurses (GSH, ASB, and EA), one psychologist (LdG) and one bioengineer (AS). The improvement experts were not involved in the evaluation of projects they had participated in with coaching or other kinds of support. In this study we explored the final reports of the improvement teams. We developed a checklist to structure the study according to the recommended improvement method (Plan- Do-Study- Act-cycles) [38], to make it easier to discuss our observations an reflect on our different assessments of the project. The criterion to be classified as successful was to document significant improvements by recognized measurement methods, based on a clear linkage between vision, aims, change efforts and measurements.

We found that 72 projects (38%) were successful, ranging from 17 to 60% within each of the eight collaboratives. A majority (78%) presented their outcomes as a shift in the level in the desired direction on a control chart.

### Data analysis

We analyzed the association between the assessments of the improvement teams (responses to 25 selected questions) and the success level of their projects. First, a logistic regression analysis was used to analyze the association between success and each of the 25 questions. Second, a factor analysis with Varimax rotation was used to identify the underlying structure of the 25 questionnaire items. Domains were extracted with an Eigen value greater than one. Kendall’s-tau-b correlation revealed that the conditions for a principal component analysis (PCA) were present. Third, when analyzing the multivariate associations between the five domains and success. Logistic regression analyses were performed, with success as the dependent variable, and the success domains as independent variables. Only domains which were significant in a bivariate analysis (defined as *p* < 0.05) were included in the multivariate regression analyses. The results from the regression analyses are presented as odds ratios with 95% confidence intervals and corresponding *p*-values. A significance level of 5% was used. All statistical analyses were performed using the software package IBM-SPSS version 21.

## Results

Our results are based on the answers to the 25 variables of the questionnaire from the successful versus the other projects. The questionnaires were returned by 53 physicians, 56 nurses, 38 psychologists, and 51 other healthcare professionals, representing 132 (70%) of the 189 improvement teams. Of the 132 responding projects, 54 (41%) had documented improvements in their final reports by recognized measure methods, and 78 (59%) had not been able to do so within the time frame of the learning collaborative (Table 2). The results of the 54 successful projects are presented in Additional file 1: Supplement 3, not as a result of this study, but to illustrate the relationship between the changes they have made, and the conditions for change reflected in our findings.

**Table 2:** Projects, completed questionnaires and response rate per success level

	Projects per success level	Filled-in questionnaires per success level	Response rate per success level
Successful projects^a^	72 (38%)	54 (41%)	75%
Other projects	117 (62%)	78 (59%)	67%
Total	189 (100%)	132 (100%)	70%

^a^The successful projects have documented improvements by recognized measure methods in their final reports

### Research question I: “What combination of what factors tend to produce “adoptable” improvement innovations?”

First, in a logistic regression analysis of the answers to the 25 questions of the questionnaire (Additional file 1: Supplement 2) we identified the variables which were significant associated with success. Two variables were found in the final model: *(*Q12) *Good guidance & help with measurement,* and (Q7) *Someone in the improvement team enjoyed working with measurement* (Table 3).

**Table 3:** Bivariate and multivariate logistic regression analysis for detection of variables significantly associated with success, (with the 25 questions as independent variables and success as dependent variable)

	Bivariate analysis				Multivariate analysis			
	OR	95% CI		*P* value	OR	95% CI		*P* value
Q12: Good guidance & help with measurement	3.17	1.82	5.52	<0.001	2.58	1.49	4.45	0.001
Q7: Enjoying to work with measurement	3.03	1.45	6.25	0.003	2.35	1.07	5.13	0.003

The table shows the independent variables in the final model, their odds ratio (OR) associated with one point increase on a 5 point Likert scale, 95% confidence intervals (CI) and *P* values

Second, to disentangle what *combination* of variables are underpinning successful improvement efforts, we performed a factor analysis of the 132 responses to the 25 questions. This analysis produced five domains: Domain I: “*Measurement and Guidance”* (nine variables), Domain II: “*Leadership engagement*”(five variables), Domain III: “*Professional environment*” (seven variables), Domain IV: “*Group process”* (two variables), and Domain V “*Leadership impact”* (two variables) (Table 4).

**Table 4:**
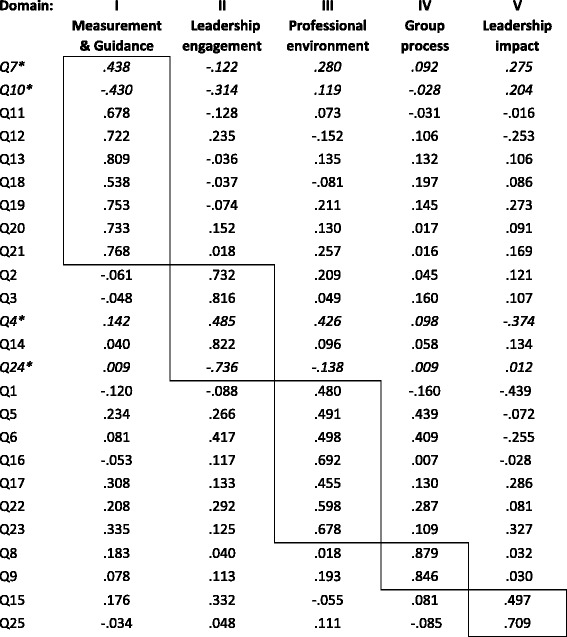
Rotated component matrix (Varimax with Kaisers normalization)

^*^One of four negative variables that are turned to positive in the other tables

Third, we studied the quartiles of the domains in the successful projects and compared the scores from the 54 successful projects with the 78 comparator projects within the five domains. Two domains were significantly associated with success: Domain I *Measurement & Guidance* (*p* = 0.002) and Domain III *Professional environment* (*p* = 0.002), (Table 5).

**Table 5:** The proportion of scores from the 54 successful projects in the five questionnaire domains

Domain	Domain I. Measurement & Guidance	Domain II Leadership engagement	Domain III Professional environment	Domain IV Group process	Domain V Leadership impact	Number of projects
Quartile^a^	Percent	Percent	Percent	Percent	Percent	*n*
I	27.3%	36.4%	25%	36%	45.8%	54
II	26.7%	35%	29.4%	37.5%	30.3%	54
III	42.9%	33.3%	57.7%	52.6%	51.2%	54
IV	64.7%	55%	52.5%	41.7%	35.5%	54
*P* value^*a*^	0.002	0.134	0.002	0.675	0.260	54 vs 78

^a^The quartiles represent the successful projects, and the *p*-values represent a Chi-square test of the 54 successful versus the 78 other projects

Finally, we made a logistic regression analysis of the five success domains. As shown in Table 6, two domains were found in the final model: “*Measurement and Guidance”* and “*Professional environment”*, confirming the findings of the crude analyses displayed in Table 5.

**Table 6:** Bivariate and multivariate logistic regression analysis of the five domains of the underlying questionnaire structure, (with the 5 domains as independent variables and success as dependent variable)

	Unadjusted analysis				Adjusted analysis			
Domain	OR	95% CI		*P* value	OR	95% CI		*P* value
I: Measurement & Guidance	3.13	1.51	5.52	0.002	2.64	1.25	5.61	0.011
III: Professional environment	3.20	1.55	6.62	0.002	2.57	1.20	5.48	0.015

The table shows the two results domains, their odds ratio (OR) associated with one point increase, 95% confidence intervals (CI) and *P* values

The complexity of our findings is displayed in Table 7 presenting the *combination* of variables that are underpinning the two success domains, illustrated by the proportion of successful and comparator projects scoring on the positive side of the scale (4 + 5) to each variable.

**Table 7:** Variables of the two success-domains and the proportion of evaluated projects scoring on the positive side of the scale (4 & 5 on a scale from 1 to 5) to each variable

Proportion of projects scoring on the positive side of the scale (score 4 and 5) to each of the variables of the success factors		
Domain I: Measurement & Guidance	Successful *n* = 54	Comparator *n* = 78
Q7: Someone in the improvement team enjoyed working with measurement	87	56
Q10: We got hold on our coach when needed between the Learning sessions (LS)	74	57
Q11: Is the availability of the coach between the LS of any importance to make successful improvements?	83	54
Q12: We had good guidance and help with measurements	81	50
Q13: Is good guidance and help with measurements of any importance to succeed with the improvement work?	59	73
Q18: The improvement team learned SPC	77	46
Q19: Is the measure method SPC of any importance to succeed with improvement efforts?	76	57
Q20: The control-charts were easy to communicate to our peers in the site.	78	51
Q21: Is it of any importance for successful changes that the control-charts are easy to communicate to the peers in the site?	67	53
Domain III Professional environment	Successful *n* = 54	Comparator *n* = 78
Q16: We based the improvement efforts on patient-focused aims	94	91
Q17: Are patient-focused aims of any importance to engage the healthcare professionals in the improvement efforts? (*No negative scores found)*	94	90
Q1: Referring to the senior expert team made our change ideas more feasible to the peers in the site	75	76
Q6: The project was well grounded in the professional environment	74	67
Q5: We organized the improvement efforts well in spite of the difficult resource situation (time and personnel)	83	83
Q22: We presented measurements continually to maintain motivation.	83	71
Q23: Is it of any importance to present measurements continually maintain motivation?	74	72

The first success domain *“Measurement & Guidance”* cover the two success variables from the first regression analysis: *Good guidance & help with measurement,* and *Someone in the improvement team enjoyed working with measurement.* In addition the findings suggest it was easier for the successful projects to get hold on their coach when needed (Q10), an availability they assessed as important to succeed (Q11). Further, did the control charts appear to be easy to communicate to their peers in the site (Q20), assessed as important when trying to make successful improvements (Q21).

*The second success domain “Professional environment”* indicate the importance of presenting patient focused aims when trying to engage of the professional environment in the improvement efforts (Q16), and the importance of presenting measurement to maintain motivation (Q23). Regardless of their success level, 83% reported they had been able to organize their improvement efforts well, in spite of a limited resource situation (Q5) (Table 7).

### Research question 2: How is the effectiveness of the continual improvement method?

Our findings reflect the intellectual underpinnings of the continual improvement method presented in Additional file 1: Supplement 1. High performing professional environments were successfully pursuing patient-centered quality improvement if they had access to a *combination* of: (a) *knowledge of best practice*, provided by professional subject matter experts, (b) *knowledge of current practice* provided by simple measurement methods, learned from (c) *improvement knowledge* experts who provided good guidance and help with measurement, and made the team able to organize the improvement efforts well in spite of the difficult resource situation (time and personnel).

## Discussion

### Our findings support improvement knowledge guidance

Our study underscores the power of good guidance and help with measurement. In contrast to most learning collaboratives abroad, The Norwegian Medical Association invested in a team of 10–15 improvement knowledge experts (coaches) to guide their improvement teams [23]. The coaching team met regularly for education and training. A system of mentoring was developed to enable experienced coaches to support the novice coaches (Additional file 1: Supplement 4).

### Our findings support measurement for improvement as a cornerstone of the project

The present study highlights the importance of using measurement to understand and reflect on the variations in current practice, and to monitor the target process continually to maintain motivation for change. Learning from the final reports of the improvement projects, the sub study indicate that successful results are connected to a clear linkage between vision, aims and proper measurements, clear and understandable improvement efforts, and the ability to communicated this all to others in an understandable way [32]. Our findings support the findings of others indicating that by measuring and monitoring variation and change with control charts, it is easier to understand and manage performance from week-to-week, communicate progress, and motivate colleagues to sustain the improvements [43–48].

### Our findings indicate common Interprofessional interest in the patients’ welfare

Our The glue for interprofessional collaboration is a common interest in the patient’s welfare, which has been emphasized as crucial by others [49]. We found that 92% of the 198 responding physicians, nurses, psychologists and other members of the improvement teams found patient-centered targets of “great” or “very great” importance for engaging their colleagues in quality improvement (Q16 Table 7).

### Our findings call for an infrastructure for improvement in healthcare

We have found that successful quality improvement efforts depend on certain conditions for change in the participating organizations that to a certain degree have been facilitated by the national learning collaborative. However, if continual improvement efforts are to become part of everyone’s work in healthcare, an infrastructure for improvement that at least is providing similar conditions for change in the local context is essential. The infrastructure should include: (a) a system that promotes leadership’s engagement at every stage of the improvement work, (b) provides easy access to clinical data needed for improvement measurement and reflection, and (c) provides qualified improvement guidance to frontline clinical improvement teams [23].

## Methodological considerations

A significant part of the overall spectrum of healthcare problems constitutes matters that are not principally biological. For this reason, it is essential to know how the philosophies of the social sciences and the biological sciences differ. One does not erroneously use the criteria for one area to judge another. The social sciences differ from the biological sciences in two aspects: They entail greater elements of overt interpretation that often enter into the collection of data. In many cases, a research result is an understanding, not an explanation. The difference between explanation and understanding however, is not as distinct as many believe [50], and in this study, we are including both.

The present study is exploring the conditions for making desired changes in healthcare. We are not reporting on a scientific experiment aiming to bring evidence to the success of the services and projects in our material. This study has been developed with the prerequisite of the known outcome of the learning collaborative projects of the Norwegian Medical Association.

Learning from high performers stems from a growing number of “positive deviance” approaches to quality improvement [50–53]. The aim of the present study is to learn from healthcare professionals in the improvement teams of those projects who have been able to document improvements based on a clear linkage between vision, aims, change efforts and measurements.

Process and outcome evaluation by improvement experts and improvement teams can illuminate the strategies and processes responsible for the improvement of the target process. In so doing, the process and outcome evaluation from sub study II [32], makes a relevant and important contribution to the development of potentially successful strategies to make positive changes in patient care [12].

It is a limitation that 75% of the projects covered by our research are from the psychiatric sector and one may conclude that the results are limited to this field. (Table 1) The general theoretical framework that we have used (see Additional file 1: Supplement 1), the findings of others (see the Background section), and the matching conditions for improvement reported by the improvement teams from the non-psychiatric settings however, does not support this limitation [23].

It is a strength that the items used in the questionnaire was based on a data collection method that invites respondents to share their point of view, rather than respond to researcher-initiated questions [54]. We designed the questionnaire to be large enough to cover the most important comments, and short enough to get a decent response rate. As described in Additional file 1: Supplement 2, this implied a step-wise reduction of the material from 233 (partly overlapping) comments to a final selection of 17. In spite of our systematic approach, we may unintentionally have excluded important comments in this process that should have been included in the study. The eight additional questions regarding the importance of the most critical incidents are meant to compensate for this limitation (Additional file 1: Supplement 2).

## Conclusion

Our findings suggest that quality improvement projects with good guidance and a sharp focus on measurement for improvement, have an increased likelihood of success.

The two success domains are well aligned with continual improvement theory. High performing professional environments were successfully pursuing patient-centered quality improvement if they had access to a *combination* of: (a) *knowledge of best practice*, provided by professional subject matter experts, (b) *knowledge of current practice* provided by simple measurement methods, learned from (c) *improvement knowledge* experts who provided good guidance and help with measurement, and made the team able to organize the improvement efforts well in spite of the difficult resource situation (time and personnel).

Our findings may be useful for healthcare organizations in the development of an effective infrastructure for improvement and thereby create necessary conditions for making quality and safety improvement a part of everyone’s job.

## Additional file

Additional file 1: Supplement QI Success Domains. (DOCX 350 kb)
