# Supplementary material for: Domains associated with successful quality improvement in healthcare – a nationwide case study
Source: BMC Health Serv Res. 2017 Sep 13;17:648. doi: 10.1186/s12913-017-2454-2 (PMC5597987; doi:10.1186/s12913-017-2454-2)
Supplement: Additional file 1: — Supplement QI Success Domains. (DOCX 350 kb) [file 12913_2017_2454_MOESM1_ESM.docx]

Supplements

To Brandrud AS, Nyen B, Hjortdahl P, Sandvik L, Haldorsen GS, Bergli M, Nelson EC, Bretthauer M.
“Domains associated with successful quality improvement in healthcare – a nationwide case study”.

Table of Contents

[**Supplement 1: The continual improvement method** 2](#_Toc487965789)

[**Supplement 2: Instrument (questionnaire) development** 3](#_Toc487965790)

[Data collection in focus groups 3](#_Toc487965791)

[Instrument (questionnaire) development 3](#_Toc487965792)

[The questionnaire 4](#_Toc487965793)

[**Supplement 3: The outcomes of the successful projects** 6](#_Toc487965794)

[Examples of control charts from the successful projects 8](#_Toc487965795)

[**Supplement 4: The improvement process guidance** 11](#_Toc487965796)

[The intellectual underpinnings 11](#_Toc487965797)

[Supplementary references 11](#_Toc487965798)

**Supplement 1: The continual improvement method**
*The Continual improvement* method of the learning collaborative is a combination of Professional knowledge and Improvement knowledge. Improvement knowledge (Suppl.1, Figure 1) implies: (1) knowledge of the system (e.g. the particular context of the place where the patient meet the provider, and the patterns of their interactions). (2) Understanding variation (with time as a variable, e.g. on a control chart). (3) The psychology of work and change (e.g. peoples’ judgements and choices). (4) Theory of knowledge (the differences in the perspectives of the people involved and how they learn). Continual improvement also implies several improvement methods and tools [22, 23, 34, 37, 39, 41, 42, 44, 45].

**Suppl.1 Figure 1: Continual improvement**


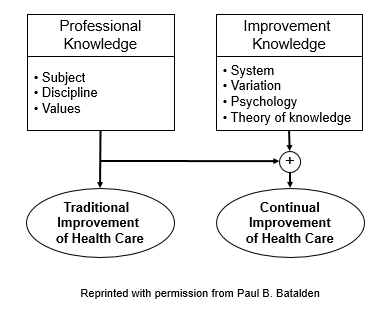


The Model for Improvement describes the systematic approach to continual improvement. The model describes a visionary approach based on Deming’s cycle, including Deming’s three fundamental questions that lead to progress: (1) What are we trying to accomplish (vision and aims). (2) How will we know that a change is an improvement (observations and measurements)? (3) What changes can we make that will result in improvement (change ideas) carried out in a Plan-Do-Study-Act cycle. The cycle illustrates that improvement should be a continual, timely and scientifically grounded process [32, 38, 42, 45].

# **Supplement 2: Instrument (questionnaire) development**

## Data collection in focus groups

Prior to the present study, critical incident data were collected in focus groups conducted by a research team of four experienced improvement advisors: one physician, two nurses, and one bioengineer. The Critical Incident Technique (CIT) was used as the data collection method, and the result was published in 2011[23]. A critical incident is one that makes a significant contribution, either positively or negatively, to an activity.[50] When seated around the table, the respondents were asked: “Think about the time when you were participating in the BTSC and the time after the project ended. What did you experience as promoting and/or inhibiting elements during and after the BTSC?” The first focus group also wanted to discuss positive spin-offs from their BTSCs. The subsequent groups were thus asked to do the same. The story telling moved around the table several times with minimal interruption by the data collectors. Their task was to support the process using active listening techniques, confirming what the respondents meant without asking other questions or providing explanations.[23, 50]

The data collectors summarized the critical incidents on flipcharts in dialogue with the respondents who assessed the accuracy of the recorded information. The focus groups lasted about 45 minutes, and each respondent reported from 5 to 20 critical incidents and positive spin offs (comments) with a mean of 12 per respondent and a total of 233 comments. A Canadian-Norwegian journalist translated the comments into English.

Instrument (questionnaire) development
Avoiding to influence the perspectives of the participants by the opinions of the improvement experts, the questionnaire was derived from a qualitative study of promoting and inhibiting elements encountered by 19 improvement team members from 16 Norwegian hospitals. We gathered the data by focus group meetings using the Critical Incident technique (CIT). The CIT is designed to evoke the perspectives of the participants’ only, without disturbing the respondents with questions based on the reflections of the improvement experts who were gathering the data.[50]

We designed the questionnaire to be large enough to cover the most important comments, and short enough to get a decent response rate. The research team started the sampling process with workshops and e-mail discussions until we had boiled down the 262 focus group comments to a representative sample of 40 questions. The instrument was citing the most critical comments, including some attitude questions about the importance of the most critical comments, and some additional background variables like profession and somatic or psychiatric care. 17 focus group comments/questions regarding critical conditions for improvement and eight questions about the *importance* of eight of the 17 conditions for change (questions) were meant for this study. A 5-point Likert response scale was tailored to each of the two kinds of questions. The instrument draft was tested on different healthcare providers with background in quality improvement efforts, and a few corrections were made.

## The questionnaire

**Conditions for improvement (n17)**

1= Strongly disagree; 2:=Somewhat disagree; 3=Neither agree nor disagree; 4=Agree; and 5=Strongly agree.

Based on your experiences as a member of the improvement team in the learning collaborative of NMA, please show your agreement/disagreement to the following focus group comments:

Q1: "Referring to the senior expert team made our change ideas more feasible to the site"

Q2: "The manager took an active part in selling in the project to the others in the site".

Q3: "The project was followed up by the leadership in the project period".

*Q4: "The leader of the improvement team had too little recognition in the site*”.*

Q5: "We organized the improvement efforts well in spite of the difficult resource situation (time and personnel)"

Q6: "The project was well grounded in the professional environment".

*Q7: "No one in the improvement team enjoyed working with measurement*"*

Q8: "We made good deals on meeting times and how we could get hold of each other between meetings"

Q9: "We had good agreements for the allocation and execution of tasks"

*Q10: "We did not get hold on our coach when needed between the Learning sessions."*

Q12: "We (the measurement responsible) had good guidance and help with measurements".

Q14: "The project was committed in the top management team".

Q16: "We based the efforts on patient centered targets".

Q18: "The improvement team learned Statistical process control (SPC)"

Q20: "The SPC charts were easy to communicate to our peers in the site"

Q22: “We presented measurements continually to maintain motivation."

*Q24:* “*The improvement efforts were not followed up by the management after the end of the project*”*

**The importance of eight of the critical conditions for improvement (n8)**

1 = No importance; 2 = Little importance; 3 = Some importance 4= Great importance 5= Very great importance

Please show your agreement/disagreement regarding what importance the different activities may have had to make successful changes.

Q11: Is the availability of the coach between the Learning sessions of any importance to make successful improvements in the participating sites (ref Q10)?

Q13: Is good guidance and help with measurements of any importance to succeed with the improvement work (ref Q12)?

Q15: Is the top management commitment to the project of any importance to succeed with the improvement efforts (Ref Q14)?

Q17: Are patient centred targets of any importance to engage the providers in the improvement efforts (ref Q16)?

Q19: Is the measure method SPC of any importance to succeed with improvement efforts (ref Q18)?

Q21: "Is it of any importance for successful changes that the SPC charts are easy to communicate to the peers in the site" (ref Q20)?

Q23: "Is it of any importance to present measurements continually to maintain motivation (ref Q22)?"

Q25: "Is this of any importance to achieve sustainable improvements (ref Q24)?"

**) We have changed negative variables (in italic) to positive when presented in Tables & Figures*

# **Supplement 3: The outcomes of the successful projects**

**Suppl.3, Table 1: The achievements of the 54 successful projects**

| **ID** | **%** | **Measurable achievements of the 54 successful projects** | Shift of level* |
| --- | --- | --- | --- |
| 1 | 31 | Reduced total Caesarean frequency from 14.2 to 9.7%, Caesarean breech from 70 to 25%, Caesarean para 0 from 19.1 to 10.2%. |  |
| 2 | 60 | Increased the use of a quality-based follow up of the mothers after an acute Caesarean from 57% to 91% of the cases. |  |
| ***3*** | ***28*** | ***Reduced mean ventilator time from 7.4 to 5.3 by improved observation & sedation. Mean stay decreased by 1 day from 9.3 to 8.3 days.***  ***(Brattebø et al. published this project in BMJ 2002; 324: 1386-1389, and the follow up results in Qual Saf Health Care 2004;13:203–205)*** | ***1*** |
| 4 | 36 | Reduced mean ventilator time from 2.9 to 6.3 by improved observation & sedation. Mean stay decreased by 2.9 days from 6.3 to 3.4 days. |  |
| 5 | 53 | Reduced the use of mechanical restraint from 23.3 to 4.6 hours per event by changing the routines. | 1 |
| 6 | 30 | Reduced aggressive outbursts per day by 30% after involving the patient in the planning and development of their own health care. | 1 |
| 7 | 35 | Reduced the length of involuntary coerced observation in the acute psychiatric ward by 35% by changing the routines. |  |
| 8 | 50 | Reduced the number of involuntary admissions (coerced observation) in the acute psychiatric ward by 50% by changing the routines. |  |
| 9 | 49 | Reduced the use of mechanical restraint by 6 hours (from 770 to 390 minutes) per event by changing the routines. Variation sign. reduced (SPC). | 1 |
| ***10*** | ***79*** | ***Increased the use of relevant information in the daily reports by a new checklist for observing serious affective disorder symptoms. (See the control chart Figure 1 below).*** |  |
| 11 | 33 | Reduced the length of stay by 33% from an average of 41.6 to 27.7 days by electroconvulsive therapy (ECT) for serious (measured) depressions. | 1 |
| 12 | 50 | Increased the patients' satisfaction with the information (41%), help (31%) and the involvement of the family in the care (50%). |  |
| 13 | 31 | Increased the number of consultations regarding bipolar disorders by 31% by a systematic staff education and training program. | 1 |
| 14 | 136 | Increased the quality of the diagnostic work by improving the medical record by 136% according to an average checklist score of 5.01 to 11.82. | 1 |
| 15 | 54 | Reduced the time from referral to diagnosis from 37 to 17 weeks. Variation reduced from 6 vs 80 weeks, to 6 vs 30 weeks. | 1 |
| 16 | 15 | Reduced the time from referral to diagnosis from 124 to 105 days. Variation (moving range) significant reduced. | 1 |
| 17 | 42 | Improved knowledge after attending a family education program about ADHD, social service and legal rights. VAS scale: 8.4, Control Group: 5.9. |  |
| 18 | 32 | Increased the quality of the diagnostic work according to a new standard from 29.5 to 39 points measured by a checklist for the medical record. | 1 |
| 19 | 69 | Reduced the time from referral to diagnosis from 236 to 74 days. Variation between neighbor observations (ref moving range chart) reduced. | 1 |
| 20 | 93 | Increased the quality of the diagnostic work according to a new standard from 5.6 to 10.8 points measured by a checklist for the medical record. | 1 |
| 21 | 30 | Reduced the duration of the diagnostic work time to diagnosis from 5.7 to 4 hours, increased comparative data collection from 70,5 to 84%. | 1 |
| 22 | 27 | Reduced the time from referral to admission from 89 to 65.25 days. | 1 |
| 23 | 44 | Reduced the time from admission to diagnosis from 136 to 76 days. Variation between neighbor observations (moving range) reduced. | 1 |
| 24 | 70 | Increased the proportion of children and adolescents being examined and care for by their primary care physician according to the ADH guidelines. | 1 |
| 25 | 30 | Reduced the total time from referral to diagnosis from 90 to 63 days for children and adolescents by a better collaboration with the pediatrician | 1 |
| 26 | 30 | Reduced the total time from referral received to diagnosis by 30% and a shift of level on a control chart after improving the diagnostic procedures. | 1 |
| 27 | 78 | Reduced the time from admission to diagnosis/decision from 87 to 19 days. Variation (moving range) significant reduced. |  |
| 28 | 52 | 205 of 392 patients had diagnosis set by a specialist after change. Specialist diagnoses increased from 4.21 per 20 weeks to 53.2 per 15 weeks. | 1 |
| 29 | 26 | Reduced the time from admission to medical treatment from 266 to 197 days, with a reduction in resource usage by 60% from 35 to 14 hours. | 1 |
| 30 | 46 | Reduced the time from admission to medical treatment from 116 to 63, earning good patient satisfaction scores, ranked as # 3 in the health region. | 1 |
| 31 | 27 | Reduced the proportion of "no-shows" from 10.3 to 7.5% by routine changes in cooperation with primary care physician, patient organizations. | 1 |
| 32 | 75 | Reduced the time from referral to admission from 20,6 to 5,2 weeks. The number of patients with this kind of rapid admission increased by 150%. | 1 |
| 33 | 46 | Reduced the time from referral to admission from 52 to 28 days. Variation between neighbor observations (moving range) reduced. | 1 |
| 34 | 35 | Reduced the time from referral to admission from 153 to 99 days, e.g. by increasing the # of 1.time admissions from 25 per 5 weeks to 78/5 weeks. | 1 |
| 35 | 70 | Increased the quality of the written hospital communication with primary care (measure checklist made by the recipient/primary care) | 1 |
| 36 | 45 | Increased the quality of the diagnostic work and improving the medical record by 45% according to an average checklist score of | 1 |
| 37 | 108 | Increased the checklist scores from 6.2 to 12.9 regarding a proper use of the research forms QRS & SRS (referral and quality of care). | 1 |
| 38 | 43 | Reduced the proportion of «no-shows" from 24 to 13.6 per week by improving the communication with patients, family and primary care. | 1 |
| 39 | 58 | Reduced the time from admission to diagnosis from 72 to30 days. Variation (moving range) significant reduced. | 1 |
| 40 | 81 | Increased the proportion of discharge summaries completed by discharge time from 3.7 to 81%, & offered 84% of those patients a summary review. | 1 |
| 41 | 71 | Increased the quality of the admission process according to a new standard from 48 to 82 points measured by a checklist for the medical record. | 1 |
| 42 | 32 | Increased the quality of the diagnostic work according to a new standard from 25 to 33 points measured by a checklist for the medical record. | 1 |
| 43 | 136 | Increased the quality of the diagnostic work according to a new standard from 3.9 to 9.2 points measured by a checklist for the medical record. | 1 |
| 44 | 200 | Increased the proportion of patients with a good, care plan documented in the medical record within the fifth consultation from 28.6 to 86.6 %. | 1 |
| 45 | 71 | Increased the quality of the individual care plan from 7 to 12 quality points measured by a checklist with quality criteria for the content. | 1 |
| 46 | 240 | Increased the quality of the diagnostic and therapeutic work from 5 to 17 quality points measured by a checklist of change criteria. | 1 |
| 47 | 97 | Reduced the time from admission to diagnosis from 72 to 2 days. |  |
| 48 | 77 | Increased the quality of the integrated care from 13 to 23 quality points according to a checklist of best practice. | 1 |
| 49 | 42 | Reduced the duration of the interprofessional diagnostic process from 29.5 to 17 days, by increasing the program compliance from 55 to 73 %. | 1 |
| 50 | 46 | Improved the diagnostic work from 8 to 11.37 points on a quality checklist, and reduced the time from decision to admission from 5 to 2 days. | 1 |
| ***51*** | ***70*** | ***Reduced time between first time referrals for first episode psychosis from 18.9 to 5.6 days, and the average age of those patients from 30 to 20 years. (see the control charts in figure 3 and 4 below)*** | ***1*** |
| ***52*** | ***64*** | ***Reduced time between first time referrals for first episode psychosis from 18.9 to 5.6 days, and referral to treatment-time from 12.44 to 10.14 days. (See the control charts in figure 2 below)*** | ***1*** |
| 53 | 40 | Reduced the time between the collaborative care efforts of BUP &VOP** regarding 16-19 year old patients from 47.33 to 28.29 days. | 1 |
| 54 | 31 | Increased the quality of family information & care from 12.14 to 15.94 points measured by a checklist of family care criteria. | 1 |

| *) Eight consecutive points or more on the desired side of the baseline median on a statistical process control chart (SPC). |
| --- |
| **) VOP=Adult mental health care , BUP= Young adult/youth mental health care |

## Examples of control charts from the successful projects

*
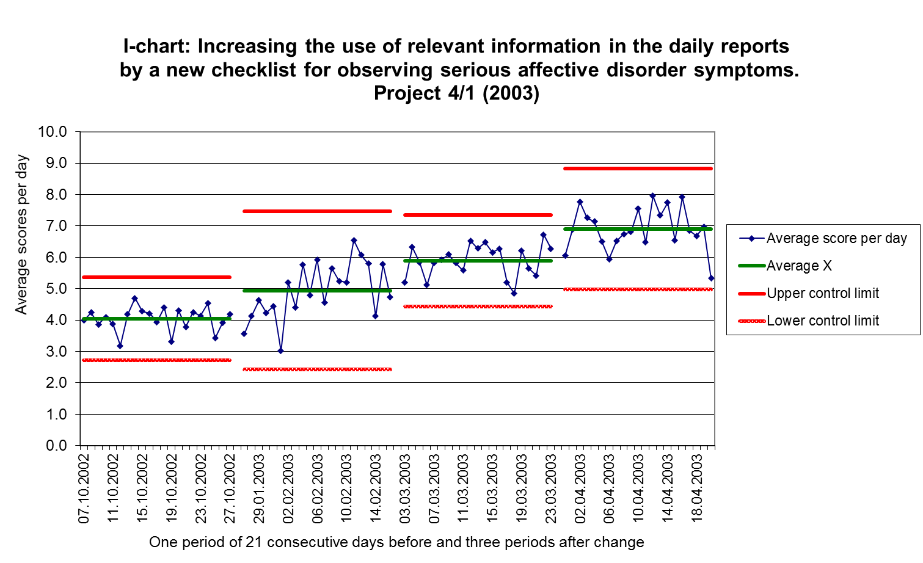
*

***Supplement 3, Figure 1; Project ID 10:*** *The improvement team aimed to Increase the use of relevant information in the daily reports by 79%. The quality of the daily reports was retrospective evaluated and scored according to a checklist of serious affective disease symptoms. The checklist was evidence based and developed by the multiprofessional improvement team in collaboration with the professional environments. Different change efforts were made to improve the providers ability to make relevant reports and the changes were tested step by step. The control chart is displaying a desired (significant) shift of level (>7 successive points above the previous (green) central line/ average) after each change period.*


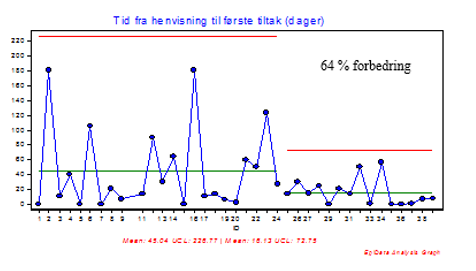


***Reduced time between referral to treatment of first episode psychosis***

***Supplement3, Figure 2; Project ID 52:*** *Figure 2: Project 8/10 (2011) Reduced time between referral to treatment of first episode psychosis from 45 to 18 days buy 64 percent. A significant shift of level achieved after change (7 successive points below the previous center line/baseline). The variation is also substantial reduced. Reduced variation is a sign of an integrated performance among the providers.*

*The barriers of the hospitals ability to respond quickly to the needs of these youngsters were substantial reduced.*


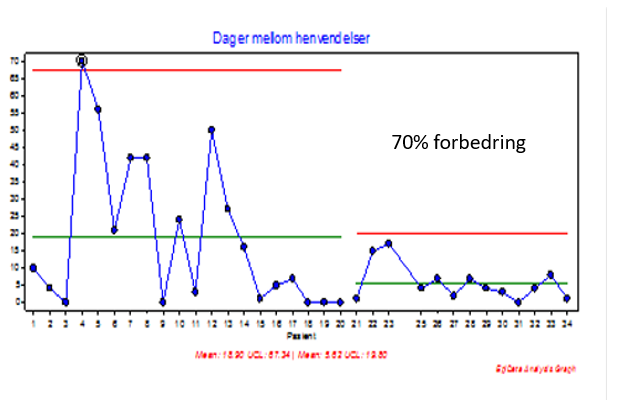


***Age of patients with first episode psychosis at first time admission***

***Reduced time between referral to treatment of first time psychosis***


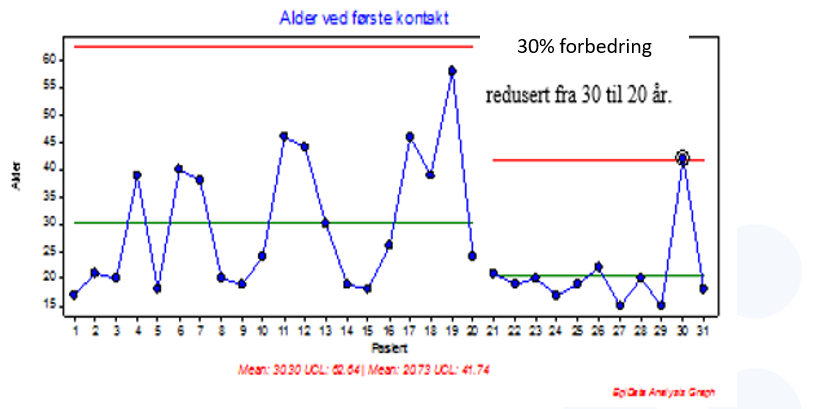


***Supplement 3, Figure 3 and 4; Project ID 51:*** *Reduced the time between first time referrals for first episode psychosis from 18.9 to 5.6 days (by 70%), and the average age of those patients from 30 to 20 years (by 30%). A significant shift of level is achieved after change in both I-charts (>7 consecutive points below baseline after change). Figure 3 display a variation that is substantial reduced, as a sign of integrated performance among the providers. Figure 4 display that a previous normal age of 42 for a first time admission is displaying a special cause variation after the intervention. That means 42 years is no longer representative for patients with a identified first time episode psychose.*

*Project 51 and 52 made several improvement efforts in collaboration with primary care, the school nurses and the teachers to help them gain a better understanding of this illness, and be able to identify the symptoms at an early stage. The barriers of the hospitals ability to respond quickly to the needs of these youngsters were substantial reduced.*

# **Supplement 4: The improvement process guidance**

## The intellectual underpinnings

We are using the term (improvement) *process* *guidance,* which is our translation of a Norwegian term that does not exist in English. But our use of term guidance is meant to cover a combination of what Caplan defines as guidance, consultation, and teaching, and what our respondents defines as help (e.g. with measurements) [51-52]. The Norwegian approach can be viewed as a combination of Schön’s “*knowing-in-action*” and “*reflection in action*”, and Eddy’s (1988) *analytic* approach. Which approach is most relevant, is determined by the situation [53-54]. *Process guidance* is rooted in Schön’s “Reflective practitioner” [53]. Tuckman’s “Five stages of group development”[55, 56], and Clarkson’s “The-team-as-a-whole” [57]. Clarkson integrated the theories of Tuckman, Berne [57, 58], Watzlawick [59] and Minuchin [60]. According to Clarkson, the role of the coach is to play the team leader good by personal guidance, without intervening in the team in a way that could take the authority away from the team leader. If joining an improvement team meeting, the coach is sitting outside the meeting table, unless the leader is inviting the coach to sit in for a while, for teaching a certain theory, showing the use of an application, how to make a control chart etc.

## Supplementary references

1. Lauvås P, Handal G. Veiledning og praktisk yrkesteori. J.W.Cappelens forlag A.s. 1994.
2. Caplan G. The theory and practice of mental health consultation. New York: Basic books 1970.
3. Schön DJ. Educating the reflective practitioner. San Francisco, Jossey-Bass 1987.
4. Eddy DM. Variations in physician practice: The role of uncertainty. I: Dowie J and Elstein A (eds): Professional judgement. A reader in clinical decision making. Cambridge 1988.
5. Tuckman BW. Developmental sequence in small groups. *Psychologic Bulletin* 1965;63:384-99.
6. Tuckman BW. Citation classic: developmental sequence in small groups.Current concerns 1984. Available at: <http://www.garfield.library.upenn.edu/classics1984/A1984TD25600001.pdf>
7. Clarkson P. Group imago and the stages of group development: a comparative analysis of the group process. *Ita News* 1988;20:4-16.
8. Berne E. The structure and dynamics of organizations and groups. New York: Grove Press 1975.
9. Watzlawick P, Weakland JH, Fisch R. Change: Principles of problem formation and problem resolution. New York, Norton 1974.
10. Minuchin SB, Fischman HC. Family therapy techniques. Cambridge: Harward 1981.
